# Therapeutic Effects and Associated Mechanisms by which “Fuyang Jiedu Huayu” Granules Treat Chronic Liver Failure in the Rat

**DOI:** 10.1155/2021/7634673

**Published:** 2021-04-17

**Authors:** Qianling Ye, Hainan Jiang, Yanmei Lan, Minggang Wang, Dewen Mao

**Affiliations:** ^1^Guangxi University of Traditional Chinese Medicine, NanNing, GuangXi 530222, China; ^2^The First Affiliated Hospital of Guangxi University of Traditional Chinese Medicine, NanNing, GuangXi 530023, China

## Abstract

**Aim:**

Fuyang Jiedu Huayu (FYJDHY) granules are a combination of five traditional Chinese medicines with known therapeutic effects against chronic liver failure (CLF). The aim of the present study was to investigate the efficacy of FYJDHY to ameliorate the effects of carbon tetrachloride- (CCl4-) induced CLF in rats and to explore the possible molecular mechanisms underlying its therapeutic efficacy.

**Methods:**

A model of chronic liver failure was established by intraperitoneal injection of 50% carbon tetrachloride into SD rats for 8 weeks. After establishing the model, rats were treated with either low-dose (4.725 kg/d), medium-dose (9.45 kg/d), or high-dose (18.9 g/kg/d) FYJDHY for 2 weeks. After treatment, samples of liver tissue and blood were harvested from rats in each group. Serum ALT, AST, and TBIL levels and prothrombin time were measured using a biochemical analyzer. The expression of Gab1 (Grb2-associated binder 1), TPO (thrombopoietin), and its receptor c-Mpl were measured using quantitative real-time PCR (RT-PCR) and Western blot analysis, and assessment of histological improvement in liver tissue was by H&E-stained tissue sections.

**Results:**

Compared with the model group, serum ALT, AST, and TBIL levels and PT of rats in the intervention group were significantly reduced (*P* < 0.05). In addition, FYJDHY alleviated pathological damage to liver tissue and increased the expression of Gab1, TPO, and its receptor c-Mpl in liver tissue, to levels statistically significant compared with the model group (*P* < 0.05).

**Conclusions:**

The therapeutic effect of FYJDHY on CLF may be related to the promotion of angiogenesis and improvement in hemopoietic function in individuals suffering from CLF.

## 1. Introduction

Chronic liver failure (CLF) is a complication of liver cirrhosis, characterized by a progressive decline in liver function and functional decompensation. In clinical practice, it is common to observe continuous malignant progressive decline in liver function as the liver decompensates due to liver cirrhosis, a continuous decrease in albumin levels, and an increase in serum bilirubin, with prothrombin activity ≤40% [1]. There are no satisfactory clinical treatments for chronic liver failure. First, the pathology of long-term cirrhosis results in the collapse of liver internal tissue structure, a reduction in liver volume, and reduced liver regeneration, causing the remaining liver cells to struggle to maintain the metabolic needs of the entire body. In addition, serious infection, hepatic encephalopathy, gastrointestinal hemorrhage, ascites, and other malignant complications are considerable challenges a patient will often encounter [2, 3]. The disease is extremely serious, with the prevention of clinical symptoms and treatment of the disease extremely difficult to achieve. Chronic liver failure is a serious complication of chronic liver insufficiency, which is generally recognized as a syndrome characterized by acute decompensation due to liver cirrhosis and organ/system failure. Liver transplantation is the only remaining choice of treatment, but there are limitations to such transplant surgery. Hepatocyte necrosis and apoptosis directly lead to the collapse of liver tissue structure, which is the principal manifestation of chronic liver failure. Therefore, improving the microenvironment for liver regeneration improves the survival rate of patients with chronic liver failure [4].

As a comprehensive medical practice characterized by holistic treatment philosophy, traditional Chinese medicine positively affects many diseases and has successfully helped Chinese individuals for more than 3000 years. In recent years, it has played an important role in the treatment of many types of liver disease, including liver failure. In traditional Chinese medicine, the basic cause of chronic liver failure is Du and Yu, with Fuyang representing the pathogenesis of chronic liver failure. Fuyang Jiedu Huayu granules (FYJDHY) (15 g of monkshood, 15 g of ginseng, 30 g of red peony, 15 g of rhubarb, and 30 g of wormwood) comprise the refined prescription formed from the new theory of Du Xie–Du Zhuo of traditional Chinese medicine of liver failure, which embodies the therapeutic concept of Jiedu, Huazhuo, and Fuyang for the prevention and treatment of chronic liver failure. In the present study, we constructed a rat model of chronic liver failure and used FYJDHY as an intervention to observe the effect of this prescription on the expression of the connexin Gab1 (Grb2-associated binder 1) [5], TPO (thrombopoietin), and its receptor c-Mpl, the key molecule for the maintenance of hematopoietic stem cell function [6].

## 2. Materials and Methods

### 2.1. Animals

Male Wistar rats, weighing 200 ± 20 g, were purchased from the Experimental Animal Center of Guangxi Medical University (Nanning, China) and were housed in a specific pathogen-free environment. Animal work was performed under certificate no. SCXK (GUI) 2014-0002. Prior to experimentation, the animals were placed in an indoor environment with controlled temperature (20–25°C) and relative humidity (55%–70%) with ventilation for 7 days to ensure adaptation. They were provided food and water ad libitum. All animal experiments were approved by the Animal Care and Use Committee of Guangxi Traditional Chinese Medicine University.

### 2.2. Drug and Reagent Preparation

**Figure 1:**
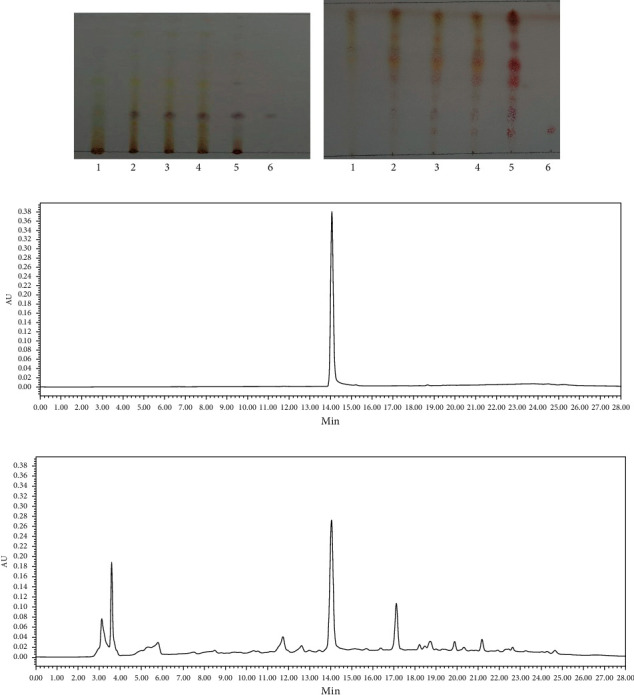
Components in Radix Ginseng, Radix Paeoniae Rubra, and Rhubarb were identified by TLC ((a)-(b)). Quality control using paeoniflorin ((c)-(d)) was conducted by HPLC. (a) 1, thin-layer chromatogram of Radix Paeoniae Rubra; 2–4, negative red peony; 5, three batches of granules; 6, control (red peony); control: paeoniflorin. (b) 1, thin-layer chromatogram of ginseng; 2–4, ginseng negative; 5, three batches of granules; 6, ginseng control herbs; ginsenoside Rb1. (c) Romatogram of paeoniflorin reference substance (1, paeoniflorin). (d) Chromatogram of Shaoyin granules (1, paeoniflorin).

FYJDHY granules were provided by Jiangyin Tianjiang Pharmaceutical Co., Ltd., batch no. 201611048, which were prepared from a single concentrated granule of a traditional Chinese medicine. The principal constituents of the FYJDHY granules were identified by thin-layer chromatography (LTC). Paeoniflorin, a representative component of herbal medicines, acting as a control for quality control purposes, was also quantified by high-performance liquid chromatography (HPLC) [7]. The results are shown in Figure 1. Carbon tetrachloride in vegetable oil was purchased from Shanghai Runcheng Biotechnology Co., Ltd., batch no. 56-23-5. Gab1 antibody (3232) was obtained from Cell Signaling Technology Company, and antibodies against TPO (ab216884) and c-Mpl (ab109003) were obtained from Abcam.

### 2.3. Instrument and Equipment

A Hitachi 7600 Automatic Biochemical Analyzer was used to measure the levels of the various biochemicals from serum. A Jung RM2015 microtome (Leica, Germany) was used for histological preparation, and H&E staining was performed using a dehydrator and spreader. Sections were observed by light microscopy (Olympus BX51T-PHD-J11, Japan). Images were analyzed using Image-Pro Plus (Media Cybernetics Company, USA).

### 2.4. Animal Groupings and Intervention

A total of 65 male SD rats were randomly divided into five groups: model (15 rats), low-dose FYJDHY granules (15 rats), medium-dose FYJDHY granules (15 rats), high-dose FYJDHY granules (15 rats), and normal (5 rats) groups. Except for the normal group, an injection of 2.0 ml/kg of 50% carbon tetrachloride vegetable oil mixture (CCl_4_ : olive oil = 1 : 1) was administered intraperitoneally three times a week (Monday, Wednesday, and Friday) for 8 weeks to construct a model of chronic liver failure [8]. Depending on the timing of the animal experiments, a 1.0 ml/kg intraperitoneal injection was administered at the end of the 9th and 10th weeks. The dosage of FYJDHY granules was calculated by the formula:

Dose_rat_ = Dose_human_ × (habeas index_rat_/habeas index_human_) × (bodyweight_human_/bodyweight_rat_) [9]. Using this formula, the medium dosage of FYJDHY granules used was 9.45 g/kg/d, equivalent to a dose in humans of 10^5^ g/60 kg/d. A low, medium, or high dose of FYJDHY (4.725, 9.45, and 18.9 g/(kg·d)) was administered by gavage as an intervention for chronic liver failure over 14 days. The normal group was administered the same volume of distilled water by intraperitoneal gavage. Gavage was conducted on the 9th and 10th weeks once a day for 2 weeks. The carbon tetrachloride vegetable oil mixture was administered by intraperitoneal injection on a Monday, Wednesday, and Friday afternoon to maintain chronic liver failure. Rats were fasted for 12 hours prior to modeling and given normal saline glucose solution to drink after. During the experimentation, the mental state, gastrointestinal symptoms, abdominal circumference, bodyweight, and rate of mortality were monitored.

### 2.5. Sample Collection and Preparation

Following drug intervention, the rats were anesthetized by injection into the peritoneum with 1-2 ml pentobarbital sodium, attached to a board, and the abdomen cleaned and disinfected with iodophor. The abdomen was cut along the midline using sterile conditions to expose the abdominal aorta from which blood was collected, placed in a centrifuge tube to clot for 30 minutes, and then centrifuged at 3000 r/min for 10 minutes to separate the serum. The liver was then dissected. The left lobe of the liver was rapidly removed and fixed in 10% neutral formaldehyde for pathological analysis. The remaining liver tissue was immediately stored at −80°C for RT-PCR and Western blot analysis.

### 2.6. H&E Staining

For histopathological examination of the liver, the tissue block was fixed for at least 24 hours to denature and aggregate protein in the tissues and cells to prevent autolysis or bacterial decomposition after cell death, maintaining the original morphological structure of the cells. Conventional paraffin embedding was performed. The tissue block was frozen in an icebox and then sectioned with a Leica RM2015 microtome to 4 *μ*m slices. Each section was sectioned onto water at 60°C for flattening prior to deposition onto glass slides. Each slide was placed onto a spreader and toaster at 37°C for drying. If required, slides were dried overnight in an oven. The sections were dewaxed twice in xylene for 5 mins each and hydrated through a gradient of ethanol concentrations: 2 mins in 100% ethanol, 1 min in each of 95%, 80% then 75% ethanol, and then in distilled water for 2 mins. Cell nuclei were stained blue by placing in hematoxylin for 5 minutes and then washed in tap water. The slides were differentiated for 30 seconds in hydrochloric acid-ethanol, soaked in tap water for 15 mins or water at 50°C for 5 mins, and then immersed in eosin solution for 2 mins to stain cell cytoplasms red. Finally, the slides were dehydrated, cleared, and sealed by placing in 95% ethanol for 1 min twice, 100% ethanol for 1 min twice, xylene carbonate (3 : 1) for 1 min, and then 2 washings in xylene for 1 min each, after which slides were sealed with neutral resin. After drying, histopathological changes in the liver were observed by light microscopy at 400x magnification in 5 visual fields that were randomly selected. Tissues underwent a pathological grading of liver injury by two professional pathologists [10].

## 3. Molecular and Biochemical Measurements

### 3.1. Detection of Serum Levels of AST, ALT, TBIL, and PT

Following collection, blood was clotted and then centrifuged at 3000 r/min for 10 mins to obtain serum. Levels of serum alanine aminotransferase (ALT), aspartate aminotransferase (AST), and total bilirubin (TBIL) and prothrombin time (PT) were measured using an automatic hemagglutination analyzer.

### 3.2. RT-PCR

TRIzol (Invitrogen, 15596-026) was used to extract total RNA from liver tissue. Integrity of the RNA was assessed by electrophoresis of 5 *μ*l RNA on a 1% agarose gel. The RNA was reverse transcribed using a TIANScript RT kit (KR104-02), in accordance with the manufacturer's instructions. PCR was performed using a 20 *μ*l reaction system (10 *μ*l 2 × SuperReal PreMix Plus, 0.6 *μ*L each of upstream and downstream primers (10 *μ*M), 100 ng cDNA, 0.4 *μ*l 50 × ROX reference dye, and the total volume made up to 20 *μ*l with RNase-free ddH_2_O) using a SuperReal premix plus SYBR Green kit. PCR was performed by heating to 50°C for 2 mins and 95°C for 15 mins and then cycling at 58°C for 30 sec and 72°C for 30 sec, for 40 cycles. Dissociation curves were plotted, and final data were analyzed using the 2^−△△^*C*_*t*_ method (Table 1).

### 3.3. Western Blot Analysis

Protein extraction was performed by homogenizing a suitable quantity of tissue in radioimmunoprecipitation assay (RIPA) buffer (Beyotime, Shanghai, China) supplemented with phenylmethylsulphonyl fluoride (PMSF, 1 mM) (Beyotime, Shanghai, China). After centrifugation at 12,000 rpm at 4°C for 10 min, the protein concentration in the supernatant was quantified using a bicinchoninic acid (BCA) protein assay kit (Beyotime, Shanghai, China). Samples were separated with sodium dodecyl sulfate-polyacrylamide gel electrophoresis (SDS-PAGE) following denaturation with sample loading buffer (Beyotime, Shanghai, China) and then transferred to polyvinylidene fluoride (PVDF) membranes. The membranes were then incubated with monoclonal antibodies against Gab1 or TPO or c-MPL rabbit anti-mouse polyclonal antibody (1 : 1000) and then sealed overnight at 4°C. Membranes were then incubated with a goat anti-rabbit IgG secondary antibody (1 : 4000, Boster Biotech, Wuhan, China). An anti-beta-actin antibody was used as the internal control. Protein bands were detected using an enhanced chemiluminescence kit (Millipore, Billerica, MA, USA) and imaged using a gel imaging system (BioRad, CA, USA). Band density was quantified using Quantity One® software.

### 3.4. Statistical Analysis

Statistical analysis was performed using a one-way analysis of variance (ANOVA) with the Bonferroni posttest analysis for normally distributed data of equal variance and nonparametric tests for other forms of data. Analysis was conducted with SPSS 22.0 software (IBM Corporation, Armonk, NY, USA). Results are presented as means ± standard deviation using GraphPad Prism statistical software version 7.0 (GraphPad, Inc., La Jolla, USA). *P* value <0.05 was considered statistically significant.

## 4. Results

### 4.1. FYJDHY Improves Liver Function in Rats with CLF

**Figure 2:**
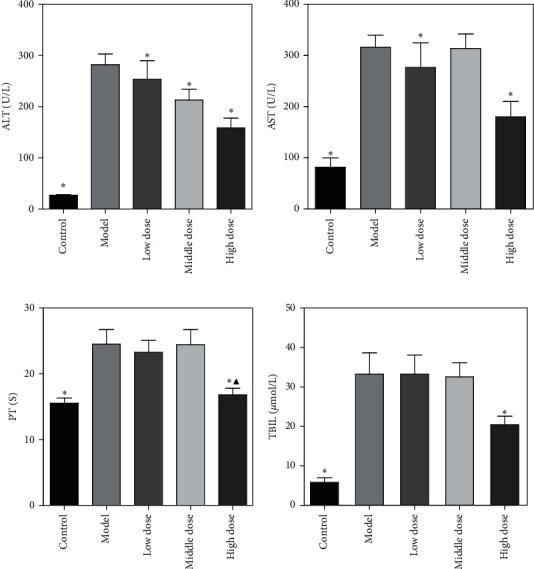
Influence of FYJDHY on AST, ALT, and TBIL levels, and PT. ^*∗*^Comparison with the model group, *P* < 0.05. ^▲^Comparison with the control group, *P* > 0.05.

At the end of the experiment, 5 rats remained in the normal group, 12 in the model group, 14 in the low-dose group, 13 in the medium-dose group, and 13 in the high-dose group. There was no significant difference in mortality in any of the chronic liver failure groups (*P* > 0.05). Compared with the normal group, levels of AST, ALT, and TBIL in serum increased significantly and PT prolonged in rats in the model group and all dosage groups (*P* < 0.05). Compared with the model group, the AST level was no different in the medium-dose group (*P* > 0.05) but different in the low and high-dose groups. ALT levels were lower in all dosage groups than in the model group, a difference that was more apparent in the high-dose group. There was a significant difference in levels of ALT between groups (*P* < 0.05). There was no difference in the TBIL level between the low or middle-dose groups and the model group (*P* > 0.05), but it was significantly lower in the high-dose group (*P* < 0.05). This indicates that bilirubin levels in the high-dose group were less elevated than in the other experimental groups. Compared with the model group, there was no difference in prolongation of the PT in the low and medium-dose groups (*P* > 0.05), whereas the difference between the high-dose and model groups was statistically significant (*P* < 0.05). There was no significant difference in PT between the high-dose and normal groups (*P* > 0.05). The results are summarized in Figure 2. Relevant results have already been published [11].

### 4.2. FYJDHY Alleviates Liver Injury in Rats with CLF

**Figure 3:**
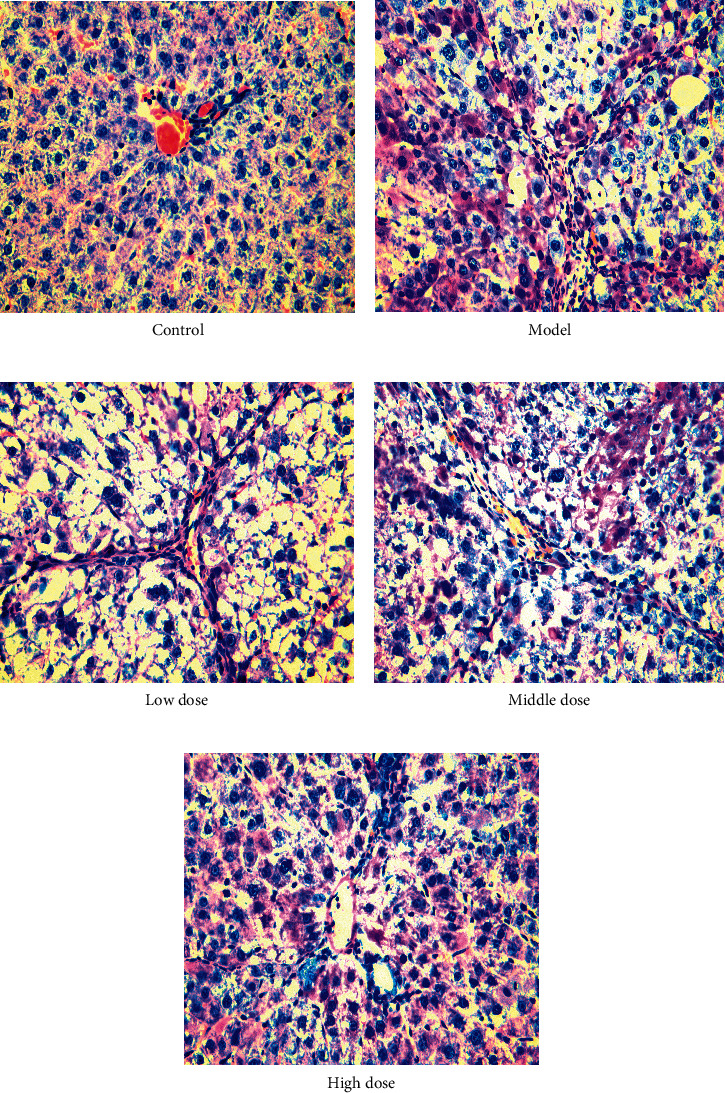
Effects of FYJDHY granules on liver histopathology in rats with acute liver failure (H&E, 400X).

Observation of the histological sections of the rats demonstrated that the lobes of the liver in the normal group exhibited no apparent signs of necrosis, injury, or fibrosis within the liver tissue. In the model group, clear signs of degeneration and necrosis of hepatocytes, infiltration of chronic inflammatory cells, proliferation of fibrous tissue, and formation of pseudolobules could be observed. After treatment with FYJDHY, liver lobule structural disruption was to some extent less apparent, with no clear turbid swelling of the liver cells, and fewer signs of necrosis than the model group, as shown in Figure 3.

### 4.3. FYJDHY Upregulates Gab1, TPO, and c-Mpl mRNA Expression in Rats with CLF

**Figure 4:**
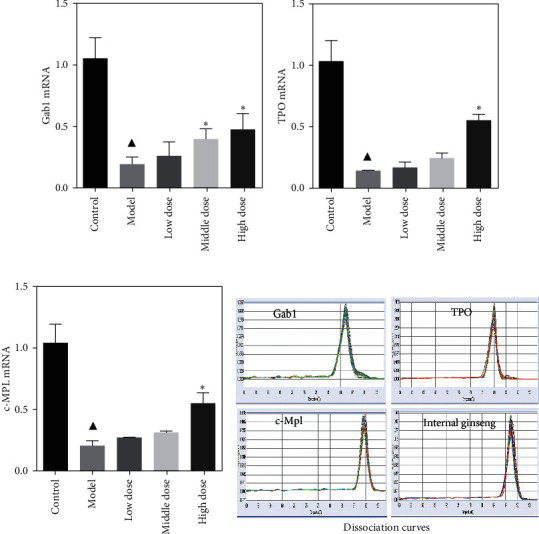
Expression of Gab1, TPO, and c-Mpl mRNA in each group. ^▲^Comparison with the control group, *P* < 0.05. ^*∗*^Comparison with the model group, *P* < 0.05.

The expression levels of Gab1, TPO, and c-Mpl mRNA in the liver tissues of rats with chronic liver failure were significantly lower than those observed in the blank group (*P* < 0.05). Treatment with FYJDHY resulted in the expression levels of Gab1, TPO, and c-Mpl mRNA being slightly higher than that of the model group, the expression of Gab1 mRNA in the medium and high-dose groups being significantly higher than that in the model group (*P* < 0.05). The difference between the high-dose and model groups was statistically significant (*P* < 0.05), as displayed in Figure 4.

### 4.4. FYJDHY Upregulates Gab1, TPO, and c-Mpl Protein Expression in Rats with CLF

**Figure 5:**
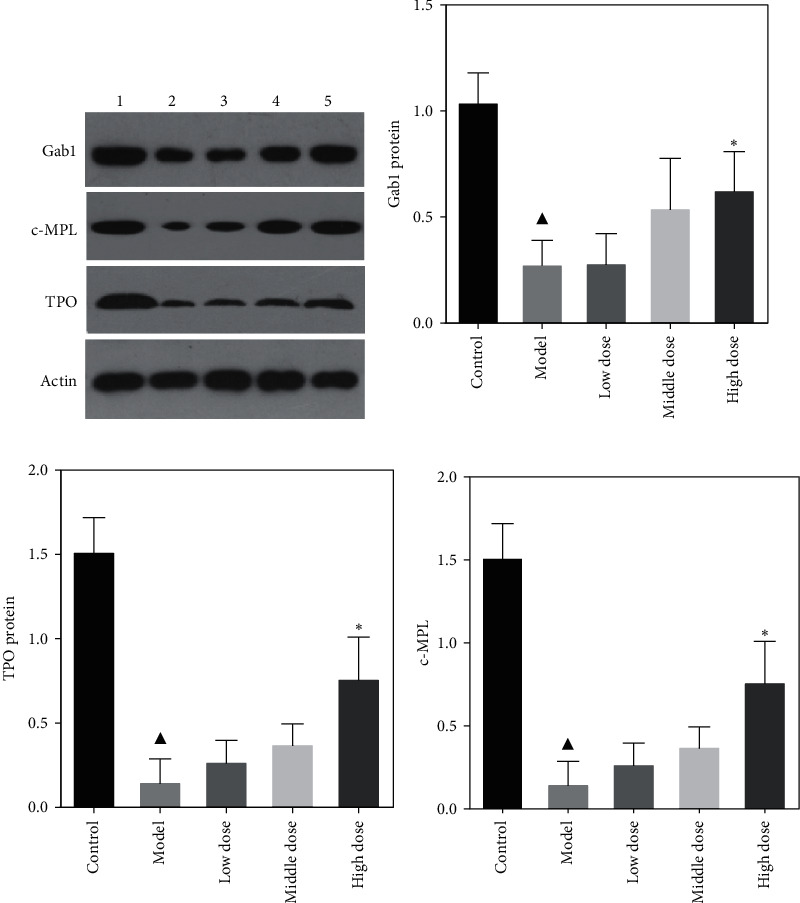
Protein expression levels of Gab1, TPO, and c-Mpl in rats from each group (first lane, control; second lane, model; third lane, low-dose; fourth lane, medium-dose; fifth lane, high-dose). ^▲^Comparison with blank group, *P* < 0.05. ^*∗*^Comparison with the model group, *P* < 0.05.

Protein expression levels of Gab1, TPO, and c-Mpl in the liver tissue of the model group were significantly downregulated compared with the blank group (*P* < 0.05). Treatment with FYJDHY resulted in higher expression levels of the three proteins, with expression patterns reflecting those of mRNA expression, especially expression of Gab1, TPO, and c-Mpl protein in the high-dose group. The differences between the model and control groups were statistically significant (*P* < 0.05), as shown in Figure 5.

## 5. Discussion

There are at present two forms of liver failure described in the academic literature: chronic and acute on chronic liver failure. The principal difference between them is the rate of decompensation of liver function and rate of disease progression [1]. Chronic liver failure and acute liver failure progress slowly, but the severity of disease is high, with difficulty in treatment and prevention, with high short-term mortality [12]. However, there are very few hepatocytes with complete functional activity in patients with chronic liver failure. In addition, during long-term pathological reconstruction of liver tissue, the original normal structure of the liver tissue collapses, causing serious microcirculation disorders [13]. On the one hand, following collapse of the structure, space and tissue structures for effective regeneration of hepatocytes do not exist, and additionally, the microcirculation disorders prevent the necessary nutritional support for effective liver regeneration. There are no effective solutions to these technical bottlenecks that hinder effective liver regeneration, the key basis for the survival of patients with liver failure. In addition, hemocytopenia (loss of red blood cells, white blood cells, and platelets) is a common symptom of liver failure [14], directly related to deterioration in liver function or disruption to the supply of nutrition and oxygen, immune status, and coagulation. At present, no more effective intervention has been defined than that of exogenous supplementation of plasma or blood cell components. The lack of improvement in microcirculation and maintenance of hematopoiesis are two technical issues preventing the clinical treatment of liver failure.

A key scientific problem to be solved is elucidation of the etiology and pathogenesis of jaundice, so that a therapeutic regimen can be formulated that improves the clinical symptoms. According to the new theory of Du Xie–Du Zhuo in liver failure (jaundice), chronic liver failure is principally caused by the interaction of Du and Zhuo with Yang Xu. Du is the root of the disease, and Zhuo is the pathological product that adds to the symptoms of the disease. Yang Xu leads to the malignant state causing withering and wasting of important organ function. Interaction of the three promotes the occurrence and development of the disease. Thus, the basic treatments Jiedu, Huazhuo, and Fuyang for chronic liver failure were proposed, namely, FYJDHY. Monkshood warms and nourishes the Yang, ginseng nourishes deficiencies, red peony activates the blood, removes stasis, and cools the blood, and rhubarb removes stasis and dredges the internal organs, while wormwood detoxifies and reduces jaundice. The entire prescription has the therapeutic effects of assisting Yang and removing toxins and turbidity, which is consistent with the etiology and pathogenesis of chronic liver failure.

Changes to liver tissue structure in CLF lead to dysfunction in blood circulation in the liver, oxygen, and nutrient deficiency, and accumulation of metabolites that further exacerbate damage to the liver cells. The degree of damage increases with duration of injury. On the one hand, a disturbance to the microcirculation directly causes ischemic injury to the tissue [15]. On the other hand, it causes a change in hemorheology leading to tissue damage. Increased blood viscosity, decreased blood cell deformability, and the formation of microthrombi can increase resistance in the circulation, even blocking the microvasculature [2]. Changes in hemorheology and the microcirculation are related and promote each other, representing a vicious circle. Disturbance to the microcirculation can directly or indirectly delay the recovery of damaged hepatocytes or exacerbate the injury, and the degree of that damage is associated with the rate of recovery of hepatocyte function and structure and the duration of recovery after reperfusion.

Gab1 is the most abundant and widely distributed Gab family protein in mammals. It can be activated by a variety of tyrosine kinase or nontyrosine kinase receptors, is stimulated by a variety of extracellular growth factors, cytokines, and a variety of T/B cell antigens, and mediates the PI3K-Akt, Crk-JNK, and Ras-MAPK signaling pathways. It has multiple biological functions, such as the promotion of cell growth, differentiation, metabolism, and the development and regulation of immunity [16]. Gab1 has been found to form a multimolecular complex with the vascular endothelial growth receptor (VEGFR) 2; it activates PLC-r and Erk1/2 through which it promotes cell proliferation, and activates Akt that promotes cell migration and survival. A reduction in the expression of Gab1 in endothelial cells leads to significantly reduced phosphorylation of FOXO1, directly affecting cell survival [17, 18]. The role of Gab1 in vascular regeneration and downstream effector molecules has been unclear in recent years. Elimination of Gab1 gene expression by gene knockout was found to prevent any regeneration of damaged vessels. Additional research has found that the principal function of the Gab1-Akt-eNOS and Gab1-PKA-eNOS signaling pathways was their respective involvement in microvascular regeneration [5].

TPO and c-Mpl, as evaluated in the present study, have been found to be associated with platelet production and may affect the chemotherapy efficacy of acute myeloid leukemia (AML) [19–21]. Other possible biological functions of TPO have not been fully revealed. Recently, it was found that TPO from liver tissue and its receptor c-Mpl play important roles in the maintenance of hematopoietic stem cell function. Specific knockout of TPO from the liver can significantly reduce hematopoietic function in experimental mice, significantly reducing the number of hematopoietic stem cells in bone marrow, while knockout of TPO expression in other organs does not appear to significantly affect hematopoietic function. Therefore, it has been suggested that TPO and its receptor c-Mpl from the liver play a key role in the maintenance of hematopoietic stem cell function [6].

In the present study, we observed that intervention using FYJDHY in a chronic liver failure model. The results demonstrated that a high dose of this drug was able to improve liver function, functioning of the coagulation system, and promotion of bilirubin metabolism, directly reflecting a reduction in the levels of AST, ALT, and TBIL and lowering of the PT in the serum of the model group. Histology indicated that the degree of liver injury after treatment with FYJDHY was less than that observed in the model group, possibly due to a reduction in liver fibrosis, improvement in microcirculation, and a decrease in necrosis and apoptosis of hepatocytes, thus protecting the hepatocytes and reducing liver tissue damage. We found that the expression of Gab1, TPO, and c-Mpl in the liver tissue of the rats with induced chronic liver failure was significantly lower than that in the normal group (*P* < 0.05), while their expression was higher following treatment with FYJDHY granules, the difference between the high-dose and the model group being statistically significant (*P* < 0.05). FYJDHY granules improved the microcirculation in tissue from rats with CLF, promoted angiogenesis, and provided the necessary space and nutritional support for liver cell regeneration, improving hemopoietic function through TPO, and alleviating many of the negative effects of bone marrow inhibition. The present study elucidated the therapeutic effects of FYJDHY in chronic liver failure at a molecular and cellular level in an animal model and represents a new treatment paradigm for chronic liver failure.

## Figures and Tables

**Table 1:** Primer sequences.

Gene	Sequence	Product length
Gab-1	GATTTTTCTGCGTTTGGGA	143 kb
	TTTCTGTCTGGCTTGAGGT	
TPO	CAGAGCCAAGATTATTCC	82
	GTGTGTCCCGTTCAGGTA	
C-MPL	CTTTCCAATTCCTCTCACCCTC	98
	CTGTCCCTGTCTCTGCCCCTCT	
*β*-Actin	CCTAGACTTCGAGCAAGAGA	140
	GGAAGGAAGGCTGGAAGA	

## Data Availability

The data used to support the findings of this study are available from the corresponding author upon request.

## References

[1] Tanaka A., Kono H., Leung P. S. C., Gershwin M. E. (2020). Recurrence of disease following organ transplantation in autoimmune liver disease and systemic lupus erythematosus. *Cellular Immunology*.

[2] Li J., Cai C., Guo H. (2015). Portal vein arterialization promotes liver regeneration after extended partial hepatectomy in a rat model. *The Journal of Biomedical Research*.

[3] Fernández-Iglesias A., Gracia-Sancho J. (2017). How to face chronic liver disease: the sinusoidal perspective. *Frontiers in Medicine (Lausanne)*.

[4] Trebicka J. (2016). Predisposing factors in acute-on-chronic liver failure. *Seminars in Liver Disease*.

[5] Lu Y., Xiong Y., Huo Y. (2011). Grb-2-associated binder 1 (Gab1) regulates postnatal ischemic and VEGF-induced angiogenesis through the protein kinase A-endothelial NOS pathway. *Proceeding of the National Academy of Sciences of the United Steates of America*.

[6] Decker M., Leslie J., Liu Q., Ding L. (2018). Hepatic thrombopoietin is required for bone marrow hematopoietic stem cell maintenance. *Science*.

[7] Gu J., Zhang H., Yao H., Zhou J., Duan Y., Ma H. (2020). Comparison of characterization, antioxidant and immunological activities of three polysaccharides from Sagittaria sagittifolia L. *Carbohydrate Polymers*.

[8] Ni S., Li S., Yang N. (2017). Deregulation of regulatory T cells in acute-on-chronic liver failure. *A Rat Model*.

[9] Zhu H., Zhang Y., Hu X. (2013). The effects of high-dose qinggan huoxue recipe on acute liver failure induced by d-galactosamine in rats. *Evid Based Complementary Alternative Medicine*.

[10] Kuznetsova D., Rodimova S., Gulin A. (2017). Metabolic imaging and secondary ion mass spectrometry to define the structure and function of liver with acute and chronic pathology. *Journal of Biomedical Optics*.

[11] Mao D., Tang N., Lan Y., Wang M. (2019). Study on the intervention of “fuyang jiedu huayu”granule on the rat model of chronic liver failure. *Shizhen National Medicine*.

[12] Ramzan M., Iqbal A., Murtaza H. G., Javed N., Rasheed G., Bano K. (2020). Comparison of CLIF-C ACLF score and MELD score in predicting ICU mortality in patients with acute-on-chronic liver failure. *Cureus*.

[13] Rutherford A., King L. Y., Hynan L. S. (2012). Development of an accurate index for predicting outcomes of patients with acute liver failure. *Gastroenterology*.

[14] Nacif L. S., Aquino F., Tanigawa R. Y. (2020). Histopathologic evaluation of acute on chronic liver failure. *Transplantation Proceedings*.

[15] Nakata M., Nakamura K., Koda Y., Kaminou T., Ogami M., Yamada R. (2002). Alterations to hepatic microcirculation in thioacetamide-induced cirrhotic livers of rats. *Osaka City Medical Journal*.

[16] Laramée M., Chabot C., Cloutier M. (2007). The scaffolding adapter Gab1 mediates vascular endothelial growth factor signaling and is required for endothelial cell migration and capillary formation. *Journal of Biological Chemistry*.

[17] Gu H., Neel B. G. (2003). The “Gab” in signal transduction. *Trends in Cell Biology*.

[18] Caron C., Spring K., Laramée M. (2009). Non-redundant roles of the Gab1 and Gab2 scaffolding adapters in VEGF-mediated signalling, migration, and survival of endothelial cells. *Cell Signal*.

[19] Li S., Shao J., Xia M. (2018). Thrombopoietin and its receptor expression in pediatric patients with chronic immune thrombocytopenia. *Hematology*.

[20] Puigdecanet E., Espinet B., Villa O. (2006). Detection of abnormalities of PRV-1, TPO, and c-MPL genes detected by fluorescence in situ hybridization in essential thrombocythemia. *Cancer Genetics and Cytogenetics*.

[21] Lorenz V., Ramsey H., Liu Z. J. (2017). Developmental stage-specific manifestations of absent TPO/c-MPL signalling in newborn mice. *Journal of Thrombosis and Haemostasis*.

